# Medical College Association

**Published:** 1879-04-20

**Authors:** 


### *MEDICAL COLLEGE ASSOCIATION.*

Professor N. S. Davis, of Chicago, President of the American Medical College Association, has appointed its next meeting at Atlanta, Ga., on Friday, May 2d, 1879, at 10 o'clock, A. M. Each Medical College in the United States is requested to send two delegates, with full power to act for their respective schools, one delegate from the Board of Trustees, and one from the Faculty.

"The object of this Association is to adopt, if possible, some uniform system of instruction, more in harmony with the requirements of the age." All the efforts of previous conventions having failed to decide upon any definite plan, the matter is still open. The meetings have not, however, been altogether fruitless of results, as they have awakened interest and discussion upon the subject. The profession at large and the county and State Associations have not yet been sufficiently aroused to the importance of the movement. We believe if the Colleges were backed and sustained by the various organized Bodies of the Profession throughout the land, that the work of elevating the standard of medical education would be promptly and successfully accomplished. Our views upon this subject have been so frequently expressed in this Journal, that we need not here reiterate them. We are in favor of progress and improvement, and ready to give our heart and hand to any movement for a higher, nobler and more dignified standard of medical education.

BOOK NOTICES.—We are forced for the want of space to omit notices of a number of interesting books and pamphlets received. They will appear in our next.

